# Tracking Progress Toward Elimination of Iodine Deficiency Disorders in Jharkhand, India

**DOI:** 10.4103/0970-0218.42061

**Published:** 2008-07

**Authors:** Binod Kumar Patro, Prasant Saboth, Saboth Zodpey, Amit Shukla, Madhu Ganesh Karmarkar, Chandrakant Sambhaji Pandav

**Affiliations:** Centre for Community Medicine, All India Institute of Medical Sciences, New Delhi – 110 029, India; 1 Department of Pediatrics, Hi-Tech Medical College and Hospital, Bhubaneswar, Orissa, India; 2 Department of Community Medicine, Government Medical College, Nagpur, India; 3 Indian Coalition for Control of Iodine Deficiency Disorder, New Delhi, India

**Keywords:** Goiter, iodine deficiency disorders, Jharkhand

## Abstract

**Research question:**

What is the current status of Iodine Deficiency Disorders (IDD) in the state of Jharkhand?

**Objectives:**

(1) To determine the status of iodine deficiency in the state. (2) To determine the availability and cost of adequately iodized salt at the retail shops. (3) To study the perceptions of the community regarding iodine deficiency, salt and iodized salt.

**Design:**

A cross-sectional community-based survey.

**Study setting:**

Thirty clusters selected through the probability proportion to size (PPS) sampling in the state of Jharkhand.

**Study participants:**

Children aged 6-12 years, households, retail shopkeepers and opinion leaders.

**Study tool:**

Quantitative and qualitative methodology using a pretested questionnaire and focus group discussion used to carry out the community-based survey.

**Results:**

Total goiter rate (TGR) was 0.9%. Median urinary iodine level was 173.2 µg/L. The proportion of individuals with urinary iodine levels less than 100 and 50 µg/L were 26.4% and 10%, respectively. Slightly less than two-thirds (64.2%) of the households were found to be consuming adequately iodized salt as measured by titration (greater than 15 ppm). Iodized salt was available across the state and the cost varied between Re. 1 and Rs. 8 per kilogram. A common belief among the community was that iodized salt is equivalent to refined packet salt that is further equivalent to expensive salt.

**Conclusion:**

The results of the present survey show that the iodine nutrition in the state of Jharkhand is optimal. Considering that the consumption of adequately iodized salt should increase from 64.2% to the goal of more than 90%, sustained efforts are required in this place to consolidate the current coverage of adequately iodized salt and increase it to greater than 90%.

## Introduction

Iodine deficiency disorders (IDD)—a term coined by Hetzel in 1983—encompasses the collective clinical and subclinical manifestations of iodine deficiency.(1) Iodine deficiency disorder impact ‘refers to all the ill-effects caused b iodine deficiency in a population, which can be prevented by ensuring adequate intake of iodine’.(2) Goiter, being the only visible manifestation of IDD, has drawn the attention of the international community. IDD encompasses abortion, stillbirths, dwarfism, deafness, squint, impaired mental function, neonatal cretinism and hypothyroidism and its complications. Iodine deficiency poses a threat to health, well-being and economic productivity of the community at large.

Iodine deficiency is a major public health problem worldwide. The available information indicate that there are nearly two billion people with iodine deficiency worldwide.(3) Though there has been substantial progress in the last decade, there remain challenges in achieving a sustainable elimination of IDD.

In India, IDD has been identified as a public health problem. Recognizing the importance of elimination of IDD as a health and developmental goal, the Government of India launched the National Goiter Control Programme in 1962; which was renamed as National Iodine deficiency Disorders Control Programme (NIDDCP) in the year 1992. A total of 263 districts out of 324 were found to be endemic for IDD (i.e. the prevalence of IDD is greater than 10%), surveyed by Director General Health Services, Indian Council of Medical Research, Medical Colleges and State Health Directorates.(4) It is estimated that more than 71 million individuals are suffering from goiter and other IDDs, while 200 million people are at risk for IDD. For the elimination of IDD in India, the main strategy adopted under NIDDCP is iodization of salt. On 17^th^ November 2005, the Ministry of Health and Family Welfare, Government of India, issued a notification banning the sale of noniodized salt for direct human consumption throughout the country under the Prevention of Food Adulteration (PFA) Act to be effective from 17^th^ May 2006.(5)

Jharkhand, erstwhile region of Bihar, was declared an independent state on 15^th^ November 2000 as the 28^th^ state of India. The state houses 26,945,829 people within 22 districts spread over an area of 79,714 km^2^. The sex ratio of the state is 941 females per 1000 males compared to the national average of 933 females per 1000 males. The state literacy rate is 53.6% that is comparable to the national average of 64.8%.(6) The state has complete ban on sale of noniodized salt for human consumption. The state is yet to set-up an IDD cell.

The objectives of the present study were the following: (1) To determine the status of iodine deficiency in the state. (2) To find out the availability and cost of adequately iodized salt at the retail shops. (3) To study the perceptions of the community with regard to iodine deficiency, salt and iodized salt.

## Materials and Methods

The methodology prescribed by the World Health Organization/United Nations Children's Fund (WHO/UNICEF)/ICCIDD(2) was used for this community-based survey. The survey was conducted between February 2006 and December 2006. All the inhabited villages and urban areas (wards in urban areas) in the state were listed along with their population. Thirty clusters were selected from this list by using a population proportion to size (PPS) technique.

School children in the age group of 6-12 years comprised the target population for the study. The children were studied by house-to-house visit. In the selected clusters, households were selected from electoral lists. In a selected household, if a target child was not available, the adjacent houses were visited till a house with a target child was found. In houses with more than one eligible child, one of them was selected using a random selection method (tossing a coin).

The sample size was calculated based on the results of the National Family Health Survey-2 (1988-89).(7) Considering the prevalence of consumption of adequately iodized salt to be 47%, absolute precision of 5%, design effect of 3 and confidence interval of 95%, the sample size calculated was 1194, which was rounded off to 1200. All attempts were made to enroll 1200 participants in the sample. A total of 40 target children were studied from each of the 30 selected clusters (40 children × 30 clusters). The survey was conducted by 3 survey teams, each covering 10 clusters. The survey teams had a two-day orientation and practical demonstration in the field in an effort to standardize the survey technique.

We prepared a pretested socioeconomic interview schedule for household and retail shop, involving components on social marketing and knowledge, attitude and practices and beliefs about IDD and iodized salt. The household questionnaire was administered to a member of the household where children were examined for the quantitative study. The interview schedule was administered by physicians in the local dialect, and the answers were translated into English and recorded. The respondent was usually a lady or head of the household who was involved in the purchase of salt. Two retail shops were selected from each cluster by purposive sampling. With regard to the qualitative component of the study, a total of 60 focus group discussions were carried out among different stakeholders, i.e., school teachers, Panchayati Raj institution members, anganwadi workers, health workers, government doctors and private practitioners.

The enlargement of the thyroid gland was examined by the physician and graded according to the WHO/UNICEF/ICCIDD classification. In case of uncertainty, the lower grade was recorded.

For the estimation of urinary iodine excretion, on-the-spot causal urine samples from all the study subjects were collected in wide-mouthed plastic bottles. The samples were transported safely to the ICCIDD reference laboratory at the Centre for Community Medicine, AIIMS, New Delhi, for the estimation of urinary iodine. Urinary iodine estimation was performed using the micropipette method.

Salt samples were collected from households and selected retail shops in sealed plastic bags and transported to the ICCIDD reference laboratory at New Delhi for the estimation of iodine in salt by the iodometric titration method.

Quality Assurance: For the intra- and interobserver variability for grading goiter, two training workshops were conducted before the actual field survey. Two supervision teams were formed, which visited 30% of the clusters to validate the data collected. The analysis of iodine levels in salt and urine samples were carried out at the ICCIDD reference laboratory. Standardized internal and external quality assurance protocols were followed.

The quantitative data were entered in an Excel spreadsheet. Descriptive statistical analysis was performed using the SPSS version 10.0. For qualitative study, the responses to open-ended questions were free listed. The main domains were identified; the individual responses were categorized and finally summarized by using qualifiers. Finally, for the open-ended questions, the observations were expressed in a semiquantitative manner.

## Results

### Quantitative component

A total of 1153 children aged 6-12 years were enrolled in the survey. There were 640 boys (55.5%) and 513 girls (45.5%) with the mean ages 8.72 ± 1.86 years and 8.67±1.98 years, respectively. The total goiter rate (TGR) was found to be 0.9%.

Urinary iodine estimation was carried out for 1121 (97.2%) children. The median urinary iodine excretion was found to be 173.2 µg/L. The urinary iodine levels in one-tenths (10%) of the samples were less than 50 µg/L, while little more than quarter (26.4%) of the samples had values less than the critical value of 100 µg/L. Table 1 presents the distribution of urinary iodine.

**Table 1:** Distribution of urinary iodine values

Median urinary iodine content (μg/L)	Number of samples	Percentage
0–19.9	41	3.7
20.0–49.9	71	6.3
50.0–99.9	184	16.4
100.0–299.9	591	52.7
≥300	234	20.9
Total	1121	100

Median Urinary Iodine Excretion = 173.2 μg/L

Iodine content from household salt samples (n = 1150) were analyzed using iodometric titration method. Among household samples, 64.2% were consuming adequately iodized salt (salt with iodine level greater than 15 ppm), while 10.8% were consuming salt with iodine content less than 5 ppm (for all practical purposes, no iodine). The iodine levels in salt samples from households ranged from 0 to 164 ppm. Table 2 presents the distribution of iodine in salt from household samples.

**Table 2:** Iodine content of salt from household sample

Iodine content (ppm)	Number of samples	Percentage
>0–4.9	124	10.8
5–14.9	288	25.0
15–29.9	436	37.9
≥30	302	26.3
Total	1150	100.0

A total of 55 salt samples from the 30 clusters surveyed across different salt types (crystal, powered, refined) were collected from retail shops and analyzed. A total of 16 (29.1%) salt samples either contained no iodine or less than 15 ppm iodine, which is considered inadequately iodized for human consumption. In the remaining 39 (70.9%) samples, the iodine content was 15 ppm or more. The price of the salt available at the retail outlets ranged from Re. 1 to Rs. 8 per kilogram.

### Qualitative Component

A total of 60 focus group discussions were carried out across different types of stakeholders i.e., school teachers, Panchayati Raj institutions members, anganwadi workers, health workers, government doctors and private doctors.

The misconception about iodized salt was noted to prevail across all groups. Among most participants from all categories of stakeholders, the common belief was that iodized salt is equivalent to the refined packet salt that is further equivalent to expensive salt. Another common finding across all groups was that all of them had some knowledge regarding the benefits of iodized salt. All of them were aware of goiter, while very few knew about the relationship between iodine and brain development.

School teachers knew about iodized salt and had some knowledge of iodine and its role in brain development. Some teachers did not know about the source or availability of iodized salt in the state.

The members of Panchayati Raj Institution were concerned about the cost of the iodized salt. Their knowledge regarding iodine deficiency and the spectrum of resulting disorders was poor.

Some of the anganwadi workers had the knowledge that the consumption of iodized salt can prevent goiter; further, many of them had the misconception that iodized salt is more clean and pure than the crystal salt. Approximately one-third of them stored the salt in closed containers and the remaining stored in open vessels and plastic jars.

Health workers (females) had the knowledge of using iodized salt for the prevention of goiter. Their knowledge regarding the role of iodine in brain development was limited. Almost all the health workers believed that crystal salt is noniodized salt and that only refined salt is iodized.

All physicians were aware of the iodine deficiency and its association with goiter. However, less than half of them knew about the spectrum of IDD. Their knowledge regarding the association between iodine deficiency and brain development was limited.

## Discussion

Geographically, Jharkhand is situated on the eastern part of the peninsula, surrounded by Bihar, West Bengal, Orissa, Chhattisgarh and Uttar Pradesh. IDD is established as a public health problem in Jharkhand, erstwhile a part of Bihar. After the formation of Jharkhand as an independent state, no comprehensive survey has been conducted to measure the IDD status in this region.

In district-wise surveys carried out by ICMR/DGHS, 8 of the 9 districts surveyed were found to be endemic for IDD, i.e., the TGR was greater than 10%.(8)

Table 3 summarizes the results of the present study. The TGR was found to be 0.9%, and median urinary iodine excretion was 173.2 µg/L, which is quite encouraging. However, slightly less than two-thirds (64.2%) of the study population were found to consume adequately iodized salt (goal > 90%). The result clearly shows that iodine nutrition in the state of Jharkhand is optimal.

**Table 3:** Status of indicators for tracking the progress toward elimination of IDD in Jharkhand

Variable	Value	Goal
Number of children enrolled	1150	Not
Applicable (NA)		
Total goiter rate (TGR)	0.9%	<5%
Number of urine samples analyzed	1121	NA
Median urinary iodine excretion (UIE)	173.2 μg/L	>100 μg/L
Proportion of values ≤100 μg/L	26.4%	<50%
Proportion of values ≤50 μg/L	10.0%	<20%
Proportion of households consuming adequately iodized salt (>15 ppm)	64.2%	>90%

The entire state has a legal provision that bans the sale of noniodized salt for direct human consumption. Salt for human consumption is mainly imported from salt-producing states such as Gujarat, Tamil Nadu and Rajasthan.

Less than one-third (29.1%) of the salt samples collected from retail shops were found to have inadequate iodine content and over one-third (35.8%) of the household population were consuming salt with iodine content less than 15 ppm, indicating that the salt is poorly iodized.

## Conclusion

The results of the present study can serve as the baseline for the IDD status for the state of Jharkhand. The tracking progress can be possible through appropriate intervention and periodic surveys. The data of this study shows that the consumption of adequately iodized salt is low (i.e., 64.2% in comparison to the goal of more than 90%), and consequently, iodine deficiency can emerge as a public health problem in future.

## Recommendation

The tracking progress using all the three indicators (Total Goiter Rate, Urinary Iodine Excretion and Salt Iodine content) should be undertaken after 3 to 5 years to sustain the efforts toward the elimination of IDD as a public health problem. There is need to focus on behavior change communication (BCC) to increase the consumption of inadequately iodized salt and sustain it thereafter. Periodic monitoring with the focus on the iodine content of salt measured on a sample basis regularly is important to ensure that the consumption of adequately iodized salt exists at the household level.
